# Mechanobiological Stimulation With Nanoencapsulated Recombinant Growth Factors Combined With Subcision for the Treatment of Complex Post-traumatic Atrophic Facial Scars: A Case Report

**DOI:** 10.7759/cureus.115763

**Published:** 2026-09-04

**Authors:** Hector Calfin, César Castro, Veronica Riquelme

**Affiliations:** 1 Research, Universidad San Sebastián, Sede de la Patagonia, Puerto Montt, CHL; 2 Aesthetic Medicine, Instituto Chileno de Rejuvenecimiento y Optimización de Medicina Estética, Santiago, CHL; 3 Dentistry, Universidad San Sebastián, Puerto Montt, CHL; 4 Faculty of Rehabilitation Sciences and Quality of Life, Universidad San Sebastián, Puerto Montt, CHL

**Keywords:** blunt cannula subcision, lipoatrophy, mechanobiology, post-traumatic atrophic scars, recombinant growth factors, stromal vascular fraction, vancouver scar scale

## Abstract

Post-traumatic atrophic scars represent a complex therapeutic challenge whose pathophysiology involves three concurrent structural deficits: reticular dermis degradation, formation of vertical fibrous tracts that anchor the skin to deep planes, and subcutaneous lipoatrophy.

We present a three-session multimodal protocol at two-week intervals, applied to a 53-year-old female patient with atrophic scars of the right hemiface secondary to a motor vehicle accident, refractory to prior reconstructive surgery, autologous fat transfer, and 10 sessions of fractional CO₂ laser. The protocol integrated tumescent hydrodissection, subcision with a 22G blunt cannula, controlled adipose tunneling to activate the stromal vascular fraction (SVF) in situ, and immediate transepidermal and retrograde delivery of nanoencapsulated recombinant human growth factors (rh-GF). Objective assessment using the Vancouver Scar Scale (VSS) demonstrated a 50% reduction in total score (from 6 to 3 points out of 14), with the greatest gains in pliability (4 → 2) and height (1 → 0), maintained at six months of follow-up. The patient spontaneously reported reduced sensation of pressure and facial stiffness, as well as improvement in her self-esteem and perceived quality of life.

This single-case report - with the limitations inherent to this study design - provides preliminary evidence that justifies the development of prospective controlled studies to evaluate the integration of mechanotransduction, adipose regeneration, and molecular signaling in complex scar defects.

## Introduction

Pathological post-traumatic atrophic scarring constitutes a condition of high clinical burden. Facial scars, in particular, generate a documented functional and psychological impact: a systematic review and meta-analysis of 21 studies reported a weighted pooled prevalence of anxiety of 26.1% (95% CI: 17.9%-36.3%) and of depression of 21.4% (95% CI: 15.4%-29.0%), with female gender (associated with anxiety), past psychiatric history, and violent traumatic etiology as associated factors [1]. Beyond the psychological impact, deep atrophic scars with an adhesive component generate functional restriction of facial movement.

Histopathologically, atrophic scars are defined by proteolytic degradation of the reticular dermis, with disorganization of the collagen architecture and loss of subcutaneous adipose tissue (local lipoatrophy) [2,3]. Recent preclinical reviews further highlight the role of fibroblast heterogeneity and the reactivation of Engrailed-1-positive fibroblasts in response to mechanical signals as proposed determinants of the scar phenotype; these findings derive from experimental in vitro and animal models rather than from established clinical evidence [3]. The depressed morphology is perpetuated by vertical sclerotic fibrous tracts connecting the damaged dermis to the muscular fascia or superficial musculoaponeurotic system (SMAS), generating negative mechanical traction [2,4]. Subcision has demonstrated efficacy in releasing these anchors [5,6]; however, its main limitation is re-adhesion during wound contraction, which leads to frequent recurrence [4,5].

The regenerative medicine paradigm postulates that effective treatment must address the three-dimensional cellular microenvironment, integrating mechanical release of fibrosis, restoration of adipose volume, and modulation of the fibroblast phenotype [2,7]. Recombinant human growth factors (rh-GF) represent a strategy to steer repair toward organized collagen deposition [4,7], and nanoencapsulated formulations - whose development in dermatology has advanced substantially in recent years [8] - allow overcoming the stratum corneum permeability barrier [9].

## Case presentation

Clinical history and background

**Figure 1:**
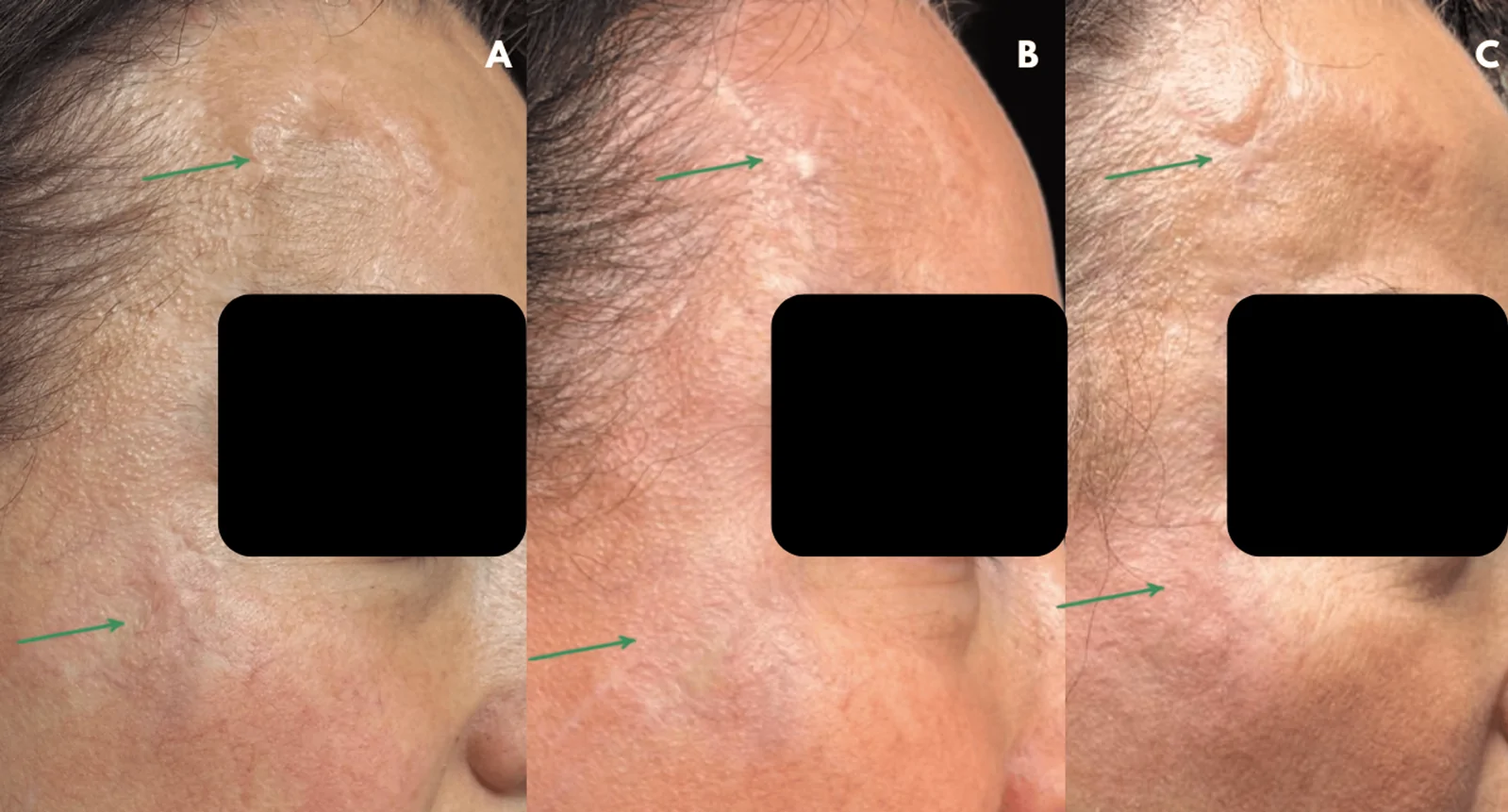
Clinical evolution of post-traumatic atrophic scars Clinical evolution of post-traumatic atrophic scars of the right hemiface at three time points, documented with standardized clinical photography (constant focal distance, illumination, and background). (A) Protocol start (Baseline, Week 0): Frontal scar with irregular texture and hypopigmentation. Erythematous malar scar with elevated relief and tissue anchoring evident. Green arrows indicate the sites of fibrotic adhesion in the frontal and malar regions. (B) End of three biweekly sessions (Week 6): Reduced malar scar relief and initial improvement of frontal texture. Green arrows show residual anchoring effects that were subsequently addressed in sessions 2 and 3. (C) Six-month follow-up: Flattened malar scar integrated into surrounding tissue. Green arrows indicate areas of successful remodeling with improved texture and elasticity, without evidence of secondary fibrosis or recurrent anchoring. The improvement of the frontal scar was clinically appreciable but is not well demonstrated photographically in this projection, whereas the malar change is clearly visible.

A 53-year-old female patient consulted for atrophic and fibrotic scars of the right hemiface secondary to blunt trauma from a motor vehicle accident five years earlier. The true pre-intervention baseline state (Figure 1A) showed scars with active erythema and vertical fibrous tracts in the frontal region, with periorbital and malar extension. Prior treatments included two reconstructive surgery interventions, one session of autologous fat transfer, and 10 sessions of ablative fractional CO₂ laser, with initial partial improvement and subsequent recurrence of the scar depression and tissue-anchoring effect. The patient was considered a candidate for this multimodal approach on the basis of atrophic scarring with a predominant adhesive (tethering) component that had proven refractory to prior surface-directed and volumetric therapies. The protocol was therefore applied as a later-line option rather than a first-line alternative to established treatments such as laser therapy (Figure 1B).

The three treatment sessions were performed on day 0 (Session 1, baseline), day 15 (Session 2), and day 28 (Session 3), at two-week intervals. The six-month follow-up assessment was conducted in April 2026. The patient provided written informed consent for the treatment protocol before the interventions; separate written informed consent for the publication of data and images for scientific purposes was obtained after the six-month follow-up was complete (Climedent SpA, publication consent form v1.0, June 2026). The June 2026 date therefore corresponds exclusively to the publication consent form and not to the treatment or follow-up period. The study was prepared in accordance with Chilean Law No. 20,584, the Code of Medical Ethics of the Chilean Medical Association, the Declaration of Helsinki (rev. 2013), the CIOMS 2016 Guidelines, and the E.J. Emanuel principles. Treatments were fully funded by the patient.

Initial clinical assessment

The scars were classified using the Goodman and Baron system - originally developed for post-acne scars but widely applied to the morphological classification of atrophic scars of diverse etiologies - as boxcar type with a predominant adhesive component (Grade 3) [10]. Objective characterization was performed using the Vancouver Scar Scale (VSS) [11]. Baseline findings were as follows: normal vascularity (0); hypopigmented pigmentation (1); banded pliability (4), with a “tight cord” consistency; and height less than 2 mm (1). The total baseline VSS score was 6 out of 14 (Table 1).

**Table 1:** Evolution of Vancouver Scar Scale (VSS) Parameters between the baseline state and six months of posttreatment follow-up. The VSS instrument and its four parameters (vascularity, pigmentation, pliability, and height) are described by Baryza and Baryza [11].

VSS parameter	Baseline	6 months	Clinical description
Vascularity	0	0	Normal - no change
Pigmentation	1	1	Persistent hypopigmentation
Pliability	4	2	Banded → Flexible
Height	1	0	< 2 mm → Flat
Total VSS	6-14	3-14	50% improvement

Biomolecular platform

The commercial platform SkinGenuity PolyGF™ (SkinGenuity LLC, Largo, FL, USA) was used, a system of high-purity rh-GF encapsulated in biomimetic lipid nanospheres smaller than 200 nm, selected specifically for the combination of factors it contains - oriented toward the four biological axes described below - and for its reproducibility versus the biological variability inherent to autologous blood derivatives such as platelet-rich plasma. It is important to note that the conceptual framework of the protocol - the sequential combination of mechanical release, adipose activation, and delivery of recombinant factors - is independent of any specific commercial product and would theoretically be replicable with other rh-GF platforms of equivalent quality. The nanoencapsulation technology aims to overcome the stratum corneum barrier (500 Dalton rule) and ensure bioavailability in the deep dermis [8,9]. The profile includes factors oriented toward four axes: extracellular matrix modulation (recombinant TGF-β3 and PDGF), angiogenesis (FGF-1, FGF-2, and HGF), cell survival and adipose support (IGF-1 and IGF-2) [12,13], and re-epithelialization (KGF, EGF, and FGF-10). The details of each factor, its International Nomenclature of Cosmetic Ingredients (INCI) nomenclature, mode of action, and biological axis are presented in Table 2. The mechanisms of action of these factors derive predominantly from animal models and in vitro studies; their transposition to the present clinical context constitutes a rational basis, not a demonstrated causal relationship.

**Table 2:** Recombinant growth factors of the biomolecular platform used, grouped by functional biological axis The described mechanisms derive predominantly from preclinical and in vitro studies. INCI: International Nomenclature of Cosmetic Ingredients; sh-: synthetic human; ECM: extracellular matrix; TGF-β: transforming growth factor beta; PDGF: platelet-derived growth factor; FGF: fibroblast growth factor; HGF: hepatocyte growth factor; IGF: insulin-like growth factor; KGF: keratinocyte growth factor; EGF: epidermal growth factor

INCI name	Growth factor	Mode of action	Biological axis	Ref.
sh-Polypeptide-5	TGF-β3	Competes with TGF-β1 for SMAD 2/3 receptors; steers collagen synthesis toward an organized basket-weave pattern rather than a fibrotic one, reducing pathological scarring.	ECM modulation	[7,12]
sh-Polypeptide-8	PDGF	Chemotaxis of fibroblasts and macrophages; stimulates mesenchymal cell proliferation and extracellular matrix synthesis during the proliferative phase.	ECM modulation	[2,13]
sh-Polypeptide-1	FGF-1 (aFGF)	Acidic fibroblast growth factor; promotes endothelial proliferation and new vessel formation (neoangiogenesis) in the repairing tissue.	Angiogenesis	[2,13]
sh-Polypeptide-11	FGF-2 (bFGF)	Basic fibroblast growth factor; potent angiogenic inducer and fibroblast mitogen; improves perfusion of the released tissue spaces.	Angiogenesis	[2,13]
sh-Polypeptide-62	HGF	Hepatocyte growth factor; pleiotropic, with antifibrotic, proangiogenic and cell-motility activity; contributes to dermal remodeling.	Angiogenesis	[13]
sh-Oligopeptide-2	IGF-1	Insulin-like growth factor 1; anti-apoptotic; promotes preadipocyte differentiation and supports subcutaneous adipose volume.	Survival/adipose support	[13]
sh-Polypeptide-31	IGF-2	Insulin-like growth factor 2; favors cell survival and adipogenesis, complementing dermal volumetric restoration.	Survival/adipose support	[13]
sh-Polypeptide-3	KGF (FGF-7)	Keratinocyte growth factor; stimulates keratinocyte migration and proliferation, accelerating re-epithelialization.	Re-epithelialization/barrier	[2]
sh-Oligopeptide-1	EGF	Epidermal growth factor; induces proliferation of basal epidermal cells and reinforces skin barrier restoration.	Re-epithelialization/barrier	[2]
sh-Polypeptide-6	FGF-10	Fibroblast growth factor 10 (KGF-2); maintains the epidermal stem cell niche and supports epithelial homeostasis.	Re-epithelialization/barrier	[2]

Instrumentation and protocol

A 22G blunt cannula was selected due to the lower abundance of subcutaneous tissue and the greater vascular proximity of the frontal and periorbital region compared to the malar region. The literature documents the use of blunt cannulas between 18G and 21G for subcision of atrophic scars, with the advantage of requiring a single entry point that reduces the likelihood of adverse events associated with multiple incisions [6]; the choice of a finer caliber (22G) in the present case responded to the need for greater anatomical safety in the periorbital region [5,6,14].

Three sessions were administered at two-week intervals - a spacing chosen to allow initial inflammatory resolution and early collagen remodeling between procedures while maintaining therapeutic continuity. The protocol consisted of three phases: Phase I - tumescent hydrodissection (2% lidocaine diluted 1:2 in 0.9% NaCl, 0.1 mL per 5 mm); Phase II - subcision with fanning and piston movements, followed by superficial subcutaneous tunneling of the released tissue planes; and Phase III - retrograde deposit of approximately 2 mL per zone into the subcision tunnels, followed by automated microneedling (1.0-1.5 mm depth) to connect the surface with the treated deep planes [8,9].

Regarding safety monitoring, only transient, self-limited erythema and edema, consistent with the expected inflammatory response to the procedure, were observed after each session; no adverse events, infection, prolonged bruising, hyperpigmentation, or scar worsening were recorded during the treatment or the six-month follow-up period.

Objective and patient-reported outcomes - six-month follow-up

Clinical evaluation was documented at three time points using standardized photography: baseline, at the end of the three sessions (week 6), and at six months of follow-up (Figure 1). Already at the end of the three sessions, reduced malar scar relief was observed, with clinically appreciated but photographically subtle improvement in frontal texture. Objective findings at six months of follow-up were most evident in the malar region and included progressive volumetric recovery of the malar atrophic depression, evident from the second session and maintained during follow-up; elimination of the tissue-anchoring effect, with resolution of facial movement restriction; textural harmonization; and absence of secondary fibrosis, with maintenance of natural elasticity (Figure 1C). VSS assessment demonstrated a reduction in score from 6 to 3 points (50% improvement), with the greatest variation in pliability (4 → 2) and height (1 → 0), stable at six months (Table 1).

Regarding patient-reported outcomes, recorded through an unstructured narrative interview - a recognized limitation compared to the prospective application of validated instruments such as POSAS 2.0 [15] or SCAR-Q [16] - the patient reported feeling “less pressure and less sensation of stiffness” and expressed that the treatment helped her “improve her self-esteem, feel more beautiful and more confident,” an account consistent with the functional and psychosocial domains identified in the literature [1]. Notably, a systematic review and meta-analysis of 30 studies (5,230 patients) reported the highest patient satisfaction rates among traumatic scars (88.3%) following scar revision, supporting the relevance of patient-reported outcomes in this specific etiology [17].

## Discussion

This report describes a multimodal protocol that addresses the three-dimensional pathophysiology of atrophic scars. The 50% improvement in VSS score, maintained at six months in a patient refractory to multiple prior interventions over five years, together with the convergence between the objective data and the patient’s subjective account, supports the clinical plausibility of the approach. Nonetheless, the single-case nature of this observation precludes establishing causal relationships or generalizing the results: this is preliminary, hypothesis-generating evidence, not confirmatory.

Proposed biological model: a mechanistic hypothesis

The conceptual framework proposed below constitutes an integrative hypothesis derived from available preclinical evidence and not a demonstrated pathway in this case. Under this model, subcision would not be merely mechanical release: cytoskeletal stretch in fibroblasts could activate integrin sensors and intracellular cascades (YAP/TAZ and Wnt/β-catenin), opening a hypothetical molecular receptivity window [18-20]; recent reviews further suggest that mechanical signals may reactivate specific fibroblast populations involved in remodeling [3]. Simultaneously, adipose tunneling would release the stromal vascular fraction (SVF), whose adipose-derived stem cells (ADSCs) secrete paracrine factors (VEGF, bFGF, IGF, and PDGF) that could attenuate pro-inflammatory TGF-β1 [13,21]. In this microenvironment, exogenous recombinant factors would act: TGF-β3, unlike TGF-β1, has shown in preclinical studies a tendency to favor collagen deposition in a basket-weave pattern [4,7,12]. Microneedling would allow nanospheres to reach the deep planes [8,9]. This model requires direct histological and molecular confirmation that the present report does not provide.

Convergence between objective and subjective outcomes

The concordance between the objective improvement in pliability (VSS 4 → 2) and the patient’s spontaneous account of reduced pressure and stiffness, maintained at six months, suggests that the outcome transcends the scale metric and reaches the everyday functional experience, the domain where epidemiology places the main burden of facial scars [1]. In future studies, the prospective application of POSAS 2.0 and SCAR-Q will allow these domains to be quantified in a standardized manner.

Extrapolation to other etiologies: scope and limits

The pathophysiology shared by atrophic scars - vertical dermal fibrosis, lipoatrophy, and mechanical anchoring - suggests varying degrees of extrapolability. Acne scars (rolling and boxcar) and post-surgical scars with adhesion represent the most direct fields. Burn scars would require protocol adaptation. Hypertrophic scars and keloids, with an opposite pathophysiology, are not candidates. All extrapolation is reasoned from a single case.

Limitations

The main limitation of this work is its single-case report nature, without a control group, which constitutes the lowest level of clinical evidence and does not allow attributing causality or ruling out confounding factors, including natural evolution or the residual effect of prior treatments. Follow-up, although extended to six months - exceeding the temporal horizon of many comparable reports - does not reach the 12 months recommended to affirm definitive durability. No histological or molecular analysis was performed to confirm the proposed biological model, which remains speculative. Patient-reported outcomes were obtained through an unstructured interview, not through validated instruments. The use of a specific commercial platform, although it does not imply a declared conflict of interest, limits direct comparison with other systems. A prospective consecutive case-series design (n ≥ 10) is proposed, with VSS + POSAS 2.0 + SCAR-Q at four time points (baseline, 3, 6, and 12 months), standardized photography, and pre- and post-treatment biopsy [22,23].

## Conclusions

This multimodal protocol was associated with a clinically meaningful, objectively measured improvement (VSS reduction from 6 to 3, maintained at six months) in a patient with complex post-traumatic atrophic facial scars refractory to multiple prior treatments. The methodological steps that were actually performed were mechanical: tumescent hydrodissection, blunt-cannula subcision, superficial subcutaneous tunneling, and delivery of recombinant growth factors. Any interpretation involving SVF activation, mechanotransduction, or paracrine molecular signaling remains an unverified biological hypothesis and is not a demonstrated component of this protocol. The patient-reported psychosocial and functional benefits should be interpreted with caution, given the high risk of confirmation bias inherent to an unstructured interview conducted by the treating clinicians. The evidence presented here is preliminary and hypothesis-generating; its value lies in justifying the design of prospective controlled studies with larger populations, validated patient-reported outcome measures (PROMs), histological endpoints, and long-term follow-up that allow the real efficacy and mechanisms of the approach to be established.
